# Carbon-Based Nanomaterials for Sustainable Agriculture: Their Application as Light Converters, Nanosensors, and Delivery Tools

**DOI:** 10.3390/plants11040511

**Published:** 2022-02-14

**Authors:** Lan Zhu, Lingling Chen, Jiangjiang Gu, Huixin Ma, Honghong Wu

**Affiliations:** 1 MOA Key Laboratory of Crop Ecophysiology and Farming System in the Middle Reaches of the Yangtze River, College of Plant Science and Technology, Huazhong Agricultural University, Wuhan 430070, China; lanzhu@mail.hzau.edu.cn (L.Z.); chenlingling@gmail.com (L.C.); mhx888999@126.com (H.M.); 2 School of Science, Huazhong Agricultural University, Wuhan 430070, China; jiangjianggu@mail.hzau.edu.cn; 3 Shenzhen Institute of Nutrition and Health, Huazhong Agricultural University, Shenzhen 511464, China; 4 Shenzhen Branch of Guangdong Laboratory for Lingnan Modern Agriculture, Genome Analysis Laboratory of the Ministry of Agriculture, Agricultural Genomics Institute at Shenzhen, Chinese Academy of Agricultural Sciences, Shenzhen 511464, China

**Keywords:** light conversion, nanofertilizers, nanopesticides, nanosensors, stress tolerance, transgenic events

## Abstract

Nano-enabled agriculture is now receiving increasing attentions. Among the used nanomaterials, carbon-based nanomaterials are good candidates for sustainable agriculture. Previous review papers about the role of carbon-based nanomaterials in agriculture are either focused on one type of carbon-based nanomaterial or lack systematic discussion of the potential wide applications in agriculture. In this review, different types of carbon-based nanomaterials and their applications in light converters, nanosensors, and delivery tools in agriculture are summarized. Possible knowledge gaps are discussed. Overall, this review helps to better understand the role and the potential of carbon-based nanomaterials for nano-enabled agriculture.

## 1. Introduction

According to an FAO report (FAO, Rome, Italy, 2020), the global production of primary crops in 2018 was 9.2 billion tons, which was about 50% higher than that in 2000. However, agricultural production is highly reliant on the use of agrochemicals such as fertilizer and pesticide, which is not sustainable. The use of pesticides and fertilizers reached 4.1 million tons and 188 million tons, respectively, in 2018 [1]. Over-use of fertilizers and pesticides not only increases the production cost of agricultural products, but also results in soil degradation and environmental pollution. Moreover, plant growth is accompanied by abiotic and biotic stresses. Thus, in addition to alleviating the heavy reliance on agrochemical applications, determining how to help plants face stress conditions is also important for efficient and sustainable agriculture. New strategies are required to address these issues to make agricultural production more efficient, resilient, and sustainable [2,3,4].

**Figure 1:**
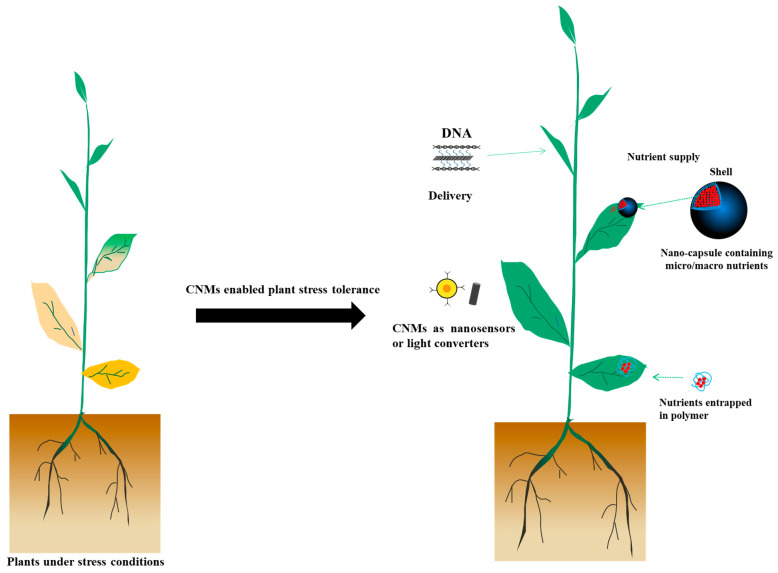
The role of CNMs in nanosensors, agrochemical delivery, and light converters in agriculture.

Nano-enabled agriculture is a rapidly growing field. Nanomaterials are materials with at least one dimension less than 100 nanometers, and have been shown to be promising in agriculture, especially in improving plant nutrition, reducing pests and diseases, improving stress tolerance, and testing the physiological state of plants. Among these materials, carbon-based nanomaterials (CNMs) are commonly used. The CNM family mainly includes carbon dots (CDs), carbon nanotubes (CNTs), carbon fullerenes (C60), graphene (GRA), graphene oxide (GO), nanohorns (CNHs), and carbon nanofibers (CNFs) [5]. The use of CNMs to improve plant stress tolerance, and thus agricultural production, is well known. For example, 10 mg/L putrescine-functionalized CDs has been used to improve grape salt tolerance [6]. Reduced GO (rGO) showed obvious inhibitory effects on *Fusarium graminearum* and *Fusarium poae* in vitro [7]. Although good reviews have been published on the use of carbon-based nanomaterials in agriculture [8], they are either focused on one type of carbon-based nanomaterial or do not mention/discuss the use of carbon-based nanomaterials in light converters, nanosensors, and delivery tools. In this review, we mainly focus on discussing the role of CNMs in nanosensors, agrochemical delivery, and light converters in agriculture (Figure 1). We hope this review can benefit the communication between the plant nanobiotechnology community and the agricultural production-related field, and can encourage people to explore more carbon-based nanomaterials for agricultural application. For more details regarding the phytotoxicity of CNMs, please refer to previous papers [9].

## 2. CNMs as Nanosenors to Detect Stress Signaling Molecules and Pesticide Residues

### 2.1. CNMs as Nanosensors to Detect Signaling Molecules in Plants under Abiotic and Biotic Stress

Against the background of climate change and global warming, the frequency and degree of plant-stress-related events have increased over time [10]. To enable sustainable agriculture, remote sensing technology was developed to monitor and manage plant stress conditions to alleviate the stress that results in the reduction in crop yield and quality [11]. Traditional remote sensing sensors mainly reveal stress through the changes in the plant phenotype and physiological characteristics during stress, which are mainly the accumulative traits [12]. This approach lacks the ability to detect the changes in chemical signaling molecules in plants upon the onset of stress. Although fluorescent dyes can be used to label signaling molecules, they have issues associated with photo bleaching, and thus cannot support long-term real-time monitoring [13]. By comparison, nanosensors may be a good candidate for monitoring chemical signaling molecules in plants during long-term and real-time tracking. Nanosensors can be used to monitor crop maturity and health, and to detect fertilizers, pesticides, and moisture in the soil, and thus can help to make quick decisions to benefit agricultural production [14]. Emerging optical nanosensors can directly detect chemical signals inside plants, providing enough time for imaging devices to detect signals and indicating the need to improve plant growth conditions [15,16].

Among nanosensors, CNTs are widely used to detect signaling molecules such as H*_2_*O*_2_* [17], Ca*^2+^* [18], and NO [19] in plants under abiotic and biotic stress, with the advantages of good fluorescence stability, long life, and emitting fluorescence in the relatively transparent near-infrared emission spectrum region of living tissues [20]. For example, the near-infrared fluorescence emitted by CNTs can be quenched by H*_2_*O*_2_* within the physiological range of 10 to 100 μM in plants, which can be used to remotely report plant stress status without causing mechanical leaf damage. Due to the sensitivity of 10 μM H*_2_*O*_2_*, hemin complexed-DNA aptamer conjugated SWCNTs were developed for real-time monitoring of H*_2_*O*_2_* content in leaves under UV, high light, and Flg22 stress [16]. This showed that the profile of leaf H*_2_*O*_2_* content changes vary among the stresses, indicating that hemin complexed-DNA aptamer conjugated SWCNTs may be a good candidate to enable early stress detection in plants. In addition, SWCNTs can be combined with a hemoglobin (Hb)-modified carbon fiber ultramicroelectrode (CFUME) to construct an in vivo H*_2_*O*_2_* sensor based on direct electron transfer. This Hb/SWCNTs/CFUME sensor can detect a dynamic range of up to 0.405 mM H*_2_*O*_2_* and has a low detection limit of 4 μM H*_2_*O*_2_* at a low working potential of −0.1 V [21]. For monitoring plant diseases such as Huanglong disease in citrus, SWCNTs functionalized with anti SDE1 (Sec-Delivered Effector 1) polyclonal antibodies can detect the SDE1 secreted protein, a biomarker of Huanglong disease, with high sensitivity and high specificity. This overcomes the traditional Huanglong disease nucleic acid and other problems of low diagnostic accuracy [22]. Single-stranded DNA-functionalized SWCNTs can also be used to detect volatile markers such as ethylhexanol, linalool, tetradecene, and phenylacetaldehyde of Huanglong disease, which is lethal to citrus, so as to realize the monitoring of the asymptomatic stage of the disease [23]. Moreover, 6-ssDNA modified SWCNTs can rapidly detect 4-ethylphenol, a major volatile organic compound released by *Phytophthora cactorum*-infected strawberries, within a few minutes via an electrostatic gating mechanism, with a wide concentration range, and high detection sensitivity and recovery [24].

Overall, these CNT-based nanosensors show potential in monitoring stress signaling molecules to better allow early detection of plant stress. However, to date, these studies have mostly been conducted in laboratories, and the proposed approaches have not been tested under actual agricultural conditions. Whether the performance of these CNT-based nanosensors will be affected by factors such as temperature (heat, cold, and freezing), wind, rain, light (high light, shading, and UV), and aerial pollutants is still unknown. It is also necessary to conduct comprehensive analysis and research on the connection between nanosensor-based sensing, plant stress detection, data storage and analysis, and agricultural equipment. Moreover, the current studies of nanosensors used in stress detection are mainly focused on the detection of stress-related plant signaling molecules, with less attention on monitoring plant nutrient deficiencies, which are also an important factor limiting agricultural production.

### 2.2. CNMs as Sensors to Detect Pesticide Residues

Pesticides enable the negative impacts of weeds and pests on crop production to be prevented and controlled, which is essential in agricultural production. However, the excessive use of pesticides leads to the release of a large number of these compounds into the environment and ecosystem. The remaining pesticide and its residues seriously endanger food safety, the ecological environment, and human health [25]. Traditional pesticide detection strategies, such as chromatography, electrophoresis, and mass spectrometry, are usually not suitable for the real-time and long-term detection of small amounts of pesticide residues and for performing on-site detection [26]. Although these methods are sensitive and accurate, they have the disadvantages of being time-consuming, and requiring complex sample preparation and expensive instrumentation. Therefore, the use of nanosensors for rapid analysis of pesticide residues may be an effective strategy. Compared with traditional methods, nanosensors have the advantages of simplicity, high sensitivity, strong selectivity, and direct on-site detection [27,28].

Nanosensors based on CNMs with autofluorescence have been widely used in pesticide detection [29]. Pesticides can be identified by quenching the autofluorescence of CDs. For example, diazinon, glyphosate, and semicarbazide residues in cherry tomatoes can be detected by CDs’ fluorescent sensors, with detection limits as low as 0.25, 0.5, and 2 ng/mL, respectively, which are the lowest detection limits of typical detection methods such as chromatography [30]. The broad-spectrum organophosphorus insecticidal malathion can also be recognized by a sensor composed of carbon dots and gold nanoparticles. The gold nanoparticles quench the fluorescence of the carbon dots based on the principle of fluorescence resonance energy transfer, but the fluorescence is detected after the detection of malathion. The intensity increases and the color changes from red to blue; thus, it is possible to quickly detect malathion through simple visual judgment [31]. Another good candidate for detection of pesticide residues is graphene quantum dots, which are widely used in biological imaging and optical sensing due to their low cytotoxicity and excellent biocompatibility [32]. For example, nitrogen-doped graphene quantum dots can be used to prepare test strip sensors for pesticides [33]. By forming a uniform PDA molecularly imprinted polymer film on the surface of a nitrogen-doped graphene quantum dot strip, thiacloprid can be specifically recognized and detected, with the detection limit as low as 0.1–4 mg/L. In addition to graphene quantum dots, graphene can also be used to prepare sensors to detect pesticides. For example, by inhibiting the peroxidase activity of graphene, aromatic pesticides can be detected by graphene doped with heteroatoms, which shows the changes in the color reaction of hydrogen peroxide and tetramethylbenzidine dihydrochloride. It is suggested that different element-doped graphene sensors can be designed to detect different aromatic pesticides [34]. Polydopamine-functionalized graphene nanozymes can effectively detect triazine pesticides by changing the activity of peroxidase through pesticides [35].

In summary, CNM-based nanosensors are good candidates for monitoring pesticide residues to benefit agricultural production. More attention on developing environmentally friendly and biocompatible CNM-based nanosensors to detect single or mixed pesticide residues is encouraged. For the use of CNMs for heavy metal detection, please refer to previous review papers [36].

## 3. CNMs as a Tool to Deliver Agrochemicals and Functional Genetic Materials

### 3.1. Use of CNMs to Deliver Agrochemicals

Use of agrochemicals, such as fertilizer and pesticide, is essential for agricultural production. However, the utilization rate of most chemical fertilizers in plants is less than 50% [37], which not only limits efficient agricultural production but also results in environment pollution. Most fertilizers are water-soluble and are easily leached in soil, resulting in environmental pollution and increased costs due to possible multiple applications of fertilizers [38]. Compared with their conventional counterparts, the efficacy gain of nanofertilizers is 18–29% higher [39]. Nanoparticles containing one or more elements required for plant growth can be directly used as nanofertilizers [40,41,42,43]. As a carrier to enable slow and/or targeted delivery of nutrients into plants, nanomaterials can also be used as nanofertilizers [44]. Regarding the use of nanofertilizers in agriculture, please refer to previous good reviews [45,46]. Here, we mainly focus on CNMs as nanofertilizers in agriculture.

Having the advantages of stable molecular structure, good biocompatibility, less toxicity, and uniform dispersion in application medium, CNMs can be used as good fertilizer carriers. For example, GO nanomaterials are good carriers of trace elements [47]. The oxygen-containing groups on its surface can electrostatically adsorb trace elements and also have two-phase release characteristics, showing quick release at the early stage, and then slow and continuous release. Indeed, GO sheets are able to deliver Zn and Cu elements more efficiently in wheat than zinc or copper salts [44]. In addition to GO, negatively charged copper CNF can also be used as a slow-release carrier of micronutrient copper [48]. CNF-Cu can transfer from roots to shoots through xylem and slowly release copper in plants, showing significantly improved water absorption capacity, germination rate, root/shoot ratio, and protein content of chicory. Negatively charged fullerenol can be used as a leaf slow-release fertilizer to promote the absorption of Fe*^2+^* in cucumber leaves and to alleviate the symptoms of iron deficiency [49]. Furthermore, previous research showed that adding nanocarbon to slow-release fertilizer can significantly improve rice yield and nitrogen use efficiency, and reduce nitrogen loss, indicating that nanocarbon can be used as an environment-friendly slow-release fertilizer coating material [50]. These results show that CNMs can be good carriers to deliver nutrients to plants or to improve the effect of fertilizers.

In addition to fertilizers, pesticides are another major component of agrochemicals for agriculture. However, public concerns exist regarding the biosafety and pollution issues of traditional pesticides due to their easy leaching, volatilization, and loss properties [50]. Excessive use of pesticides has also caused many problems that need to be addressed urgently, such as plant disease resistance, destruction of soil biodiversity, and adverse effects on human health and the environment [51]. Therefore, more efficient and environmentally friendly solutions regarding the use of pesticides are encouraged. Nano-pesticides (including nano-insecticides, nano-herbicides, and nano-fungicides) can reduce volatilization and degradation of pesticides, improve utilization efficiency, reduce the use of pesticides, and alleviate environmental risks [52,53]. In addition to adsorbing harmful organic matter to reduce the solubility and bioavailability of organic matter, CNMs are promising materials that can be used as a pesticide carrier to improve the utilization efficiency of pesticides [54,55,56,57]. Currently, due to the unique physical and chemical properties mentioned above, GO is one of the most widely used carriers in the field of nano-pesticides. For example, rGO has the advantages of high pesticide adsorption capacity (up to 1200 mg/g for chloropyrifos), low toxicity, good antibacterial performance, insensitivity to pH value change, and the ability to be reused [57]. GO loaded with red spider insecticide can be completely adsorbed on the surface of the red spider and have a strong toxic effect [58]. GO can carry different types of pesticides through surface modification. Tong et al. (2018) used polydopamine-modified GO as the carrier of water-soluble pesticides, which alleviated the issue of easy loss of water-soluble pesticides, enabled controlled release of pesticides, enhanced the adhesion between pesticides and plants, and thus improved the utilization efficiency [59]. Hydrophobic pesticides can be loaded by polylactic acid-modified GO [60]. However, GO also has disadvantages, such as low stability under acidic solution [61]. Song et al. (2019) developed nano-biochar as the carrier of emamectin benzoate, and used carboxymethyl chitosan as the pH-responsive switch to control the delivery and release of emamectin benzoate. As a result, the water solubility, dispersion stability, and UV resistance of the delivery system were significantly improved, ensuring its long-term control of pests [62]. In addition to GO, CNTs can also be used as a sustained-release system for pesticides. For example, MWCNTs grafted with polycitric acid (PCA) can deliver zineb, an antifungal pesticide. Compared with zineb in bulk, the novel CNT-PCA-Zineb hybrid material has better water solubility, higher stability, and stronger toxicity to Alternaria [63].

Overall, CNMs are good candidates to deliver agrochemicals into plants with better efficiency than conventional fertilizers and pesticides. However, there is an urgent need to understand how plants respond to their exposure. Moreover, the addition of CNMs has increased the complexity of the agro-ecosystem; whether they represent a new pollutant or a new opportunity is discussed in detail by Kah [64]. With regard to the future of nano-agrochemicals, it is necessary to fully consider the views in many fields of science, industry, and regulation, so that the agrochemicals sector can make use of nanotechnology and reduce its negative impact on human beings and the environment as much as possible.

### 3.2. Use of CNMs to Deliver Functional Genetic Materials

To feed an expected population of over 9 billion in 2050, the breeding of stress-tolerant species is of importance to ensure the food supply in the near future. At present, the commonly used transformation methods in plants, such as gene gun bombardment and agrobacterium-mediated transformation, have problems such as restriction of the host type, restriction of transformation efficiency by the cell wall, damage to the plant tissue, and pathogenicity [65]. In recent years, nano-enabled transgenic events have shown convincing progress. Among the nanomaterials used, carbon nanotubes and carbon dots are important. Compared with agrobacterium-mediated gene transformation, nanomaterials can deliver nucleic acids faster. In addition, some auto-fluorescent CNMs, such as CDs, can form relatively small complexes with nucleic acids and deliver the nucleic acids into cells, and track the complexes directly and conveniently [65,66,67]. A PEI-modified CD-siRNA delivery system has been developed to deliver and silence target genes in tobacco and tomato [68]. In addition to CDs, CNTs are the most widely tested carriers for nano-enabled transgenic events. Demirer et al. (2019) established a method for delivering plasmid DNA with PEI-modified SWCNTs, which were able to effectively deliver plasmid DNA to wheat and cotton without transgene integration, and showing highly expressed YFP protein [69]. Further studies showed that SWCNTs can also deliver siRNA to mature plant leaves to achieve instantaneous gene silencing, with a gene knockout efficiency as high as 95% [70]. In addition, SWCNTs modified with chitosan can achieve chloroplast genetic transformation without external biological or chemical assistance, which is much cheaper and simpler than conventional methods [71]. Researchers also developed a system of SWCNTs and conventional cell-shuttle peptides to improve the efficiency of targeted peptide–plasmid transformation [72]. Overall, CNMs play an important role in nano-enabled transgenic events and may be good candidate for targeted delivery of functional materials. However, the plant transformation method based on CNMs is still at the embryonic stage and needs to be further explored.

## 4. CNMs as a Light Converter for Augmenting Plant Photosynthesis

Plants convert solar energy into chemical energy through chloroplast photosynthesis [73,74]. However, the utilization rate of sunlight by chloroplasts is less than 10%, and is limited to the visible spectral range (400–700 nm), mainly in the blue and red regions [75,76]. UV and nIR lights are not utilized for plant photosynthesis. Therefore, to expand the light spectrum for plant photosynthesis, developing high-performance light conversion materials with blue and red broadband emissions to make maximum use of solar energy may be a feasible approach [77,78,79,80].

### 4.1. CNMs as a Down-Conversion Light Converter

UV light (200–400 nm) induces the generation of reactive oxygen species in plants and negatively affects agricultural production [81]. In order to increase plant photosynthesis, various light-trapping nanomaterials have been used to convert poorly absorbed ultraviolet light into highly absorbed visible light to improve the conversion efficiency of the light used by chloroplasts [82]. Due to their stable emission and adjustability of the photoluminescence spectrum, CNMs are widely used as down-conversion nanomaterials (DCNMs) to convert ultraviolet (UV) light into photosynthetic active radiation [83,84,85,86,87]. Most CDs synthesized at present can emit blue fluorescence under UV excitation [88,89,90]. Vinyl alcohol-encapsulated CDs converted UV to blue light and enhanced photosynthetic efficiency in lettuce [91]. Amine-functionalized CDs can be strongly conjugated on chloroplast surfaces and promote photosynthesis by accelerating the conversion of solar energy [92]. Li et al. (2018) designed a new dual-wavelength luminescent CD that exhibits strong absorption in the UV light region and emits light that exactly matches the chloroplast absorption spectrum (blue and red light) [93]. The adenosine triphosphate produced by the hybrid photosystem (chloroplast coating with CDs) in vitro is 2.8 times that of the chloroplast itself. In the in vivo experiments, the electron transfer rate of the Rome lettuce leaves coated with CDs increased by 25% at the maximum. It should be noted that the impact of CDs on plant photosynthesis may be affected by the quantum yield. For example, chloroplasts can only use the blue fluorescence re-emitted by CDs with medium QY (46.42%) to enhance photosynthesis, but not low and high quantum yield CDs [94]. This suggests that properties such as the emission intensity, quantum yield, and emission light spectrum of CNMs used for converting UV to visible light should be properly designed before being applied to plants. Moreover, as a UV-visible light color converter, CDs can also be applied to plastic films and LEDs for greenhouse to promote plant growth [95,96,97,98].

### 4.2. CNMs as an Up-Conversion Light Converter

nIR light accounts for about 52% of the solar spectrum, but cannot be used by plants, resulting in a serious waste of solar light resources [99]. Up-conversion nanomaterials (UCNMs) can convert nIR light to visible light that can be utilized by plants. The conversion of nIR to visible light by UCNMs is a nonlinear optical process, which absorbs two or more low-energy photons from nIR light and converts them into high-energy photons having a shorter wavelength and stronger energy via energy transfer, excited state absorption, or multiphoton absorption [100]. Carbon nanomaterials can work with up-conversion optical materials to promote nIR light conversion to enhance plant photosynthesis [101,102]. Doping CDs into the up-conversion material NaYF4: Yb, Er, CDs can shift the green emission of NaYF4: Yb, Er to red light [103]. Mung beans sprayed with NaYF4: Yb, Er@CD nanocomposite showed a significantly increased photosynthetic rate [103]. It is argued that CNMs can also be used as up-conversion nanomaterials (UCNMs). However, to date, CNMs as UCNMs have rarely been directly applied to the improvement in plant photosynthetic efficiency.

In summary, the use of CNMs to convert UV and nIR to visible light may be a potential means to augment plant photosynthesis. However, to date, the leaf and/or root application of CNMs as light conversion materials to improve plant growth is still mainly at the proof-of-concept stage. Moreover, to facilitate the use of CNMs as a light converter in plants, addition factors need to be considered, such as (1) the biocompatibility, long-term safety, and toxicity of light converting CNMs in plants; (2) the light conversion efficiency; and (3) the heat generated during CNM-enabled light conversion in plants. For example, after nIR dyes are attached to the surface of lanthanide-doped UCNPs, the up-conversion efficiency is more than 30,000 times higher than that of UCNPs alone [104]. Moreover, some optical nanomaterials, such as carbon nanohorns (CNHs) and gold nanorods (AuNRs), generate local heat through photothermal conversion under nIR laser irradiation [105,106].

## 5. Concluding Remarks and Perspective

In this paper, the applications of CNMs in agricultural production are discussed with the emphasis on their usage as biosensors, carriers, and light convertors. It is clear that carbon nanomaterials may play an important role in future agricultural production, including in increasing food production and helping with the sustainability of agriculture. However, their effects may vary depending on factors such as plant species, CNM type, and dosages. At present, the research into CNMs in agriculture is mostly limited to the laboratory, so it is necessary to obtain a large quantity of field application data to allow their future large-scale application in agriculture.

## Data Availability

The data is contained within the article.
